# Influence of ocular biometric factors on the defocus curve in an enlarged depth-of-focus intraocular lens

**DOI:** 10.1007/s10792-022-02496-y

**Published:** 2022-09-27

**Authors:** Galadriel Giménez-Calvo, Francisco de Asís Bartol-Puyal, Irene Altemir, Silvia Méndez-Martínez, Cristina Almenara, Diana Soriano-Pina, Ane Murueta-Goyena, José Manuel Larrosa

**Affiliations:** 1 Ophthalmology Department, Miguel Servet University Hospital, Paseo Isabel la Católica, 1-3, 50009 Zaragoza, Spain; 2 Miguel Servet Ophthalmology Research Group (GIMSO), Aragón Institute for Health Research (IIS Aragón), Zaragoza, Spain; 3 University of Zaragoza, Zaragoza, Spain; 4 Neurodegenerative Diseases Group, Biocruces Bizkaia Health Research Institute, Barakaldo, Bizkaia Spain; 5 Department of Neurosciences, University of the Basque Country (UPV/EHU), Leioa, Bizkaia Spain

**Keywords:** Multifocal IOLs, EDoF IOLs, Diffractive, Cataract, Quality of vision

## Abstract

**Background:**

To assess the influence of biometric measurements on the defocus curve after the implantation of enlarged depth-of-focus (EDoF) intraocular lens (IOL).

**Methods:**

Patients who underwent cataract surgery with bilateral implantation of Tecnis Symfony IOL were enrolled. Preoperatively, axial length (AL), corneal keratometry (K), pupil size and corneal aberrations were measured. 1 month after surgery, distance, intermediate, and near visual acuities (VA) were recorded. At 3 months, monocular and binocular corrected contrast sensitivities under photopic and mesopic lighting conditions were measured with CSV-1000E test. At 6-months, the defocus curve between −5.00 to + 3.00 diopters (D) was assessed in steps of 0.50 D, and NEI-RQL-42 questionnaire was administered.

**Results:**

One hundred thirty one eyes of 66 patients were included. Binocular logMAR VA better than 0.1 for intermediate vision was obtained in 90% of patients, whereas only 17.7% obtained that result in near vision. The rate of satisfaction was high (96%) and most of them (85.5%) had no or little difficulties in near vision. The mean amplitude of the defocus curve was 2.35D ± 0.73D, and smaller AL, smaller pupils, younger age, and male sex were associated with wider range of clear vision.

**Conclusions:**

Tecnis Symfony IOL enables functional vision at all distances, but demographic variables and preoperative biometric measurements like AL and pupil size influence the postoperative amplitude of the defocus curve. These parameters could be used to predict the performance of EDoF IOLs.

## Value statement

What was known

- Clinical efficacy and safety of extended depth of focus (EDoF) IOLs after cataract surgery has been proven.
- Visual capabilities in near vision are usually limited to 1 m in patients implanted with EDoF IOLs, although outcomes vary from patient to patient, some subjects achieving excellent visual acuities (≤ 0.1 LogMAR) in near vision.
- Ocular biometric parameters are known to influence on the range of clear vision with multifocal IOL, but to our knowledge, no prior study has evaluated this relationship in EDoF IOLs.

What this paper adds

- Bilateral implantation of EDoF IOLs provides satisfactory visual results in highly selected patients.
- Demographic and preoperative biometric measurement are associated with the amplitude of the defocus curve, even after controlling for the effect of age and sex.

## Introduction

Although monocular intraocular lenses (IOLs) have improved the visual quality of patients undergoing cataract surgery, the independence of correction for intermediate and near vision is increasing. In the last decade, several types of multifocal IOLs have been designed to improve spectacle independence after cataract surgery. Unlike the preceding monofocal IOLs, multifocal IOLs provide good visual outcomes at different distances [1]. Moreover, new generation multifocal IOLs have been designed to overcome some disadvantages of previous multifocal IOLs attributable to their inherent optical design, such as the perception of photic phenomena, reduced contrast sensitivity (CS), and decreased visual function in dim light environments [2, 3]. For a successful outcome and meet patients' expectation, it is crucial to consider preoperative factors, including biometric measurements, pupil reactivity or patient’s lifestyle [1].

Tecnis® Symfony® ZXR00 (Abbott Laboratories, Illinois, USA) produces an extended depth of focus in order to improve visual outcomes at intermediate distances. Previous studies have revealed that Tecnis Symfony IOLs exhibit good visual outcomes after surgery [4, 5]. In particular, they provide better objective and subjective quality of vision and CS compared to trifocal lenses and produce less photopic phenomena [5–7]. Nevertheless, their performance is worse at near vision, and acceptable intermediate vision varies largely among patients. Therefore, the aim of this study was to analyze demographic and preoperative factors related to enlarged postoperative depth-of-focus in patients implanted with Tecnis Symfony ZXR00.

## Methods

### Study design and participants

This prospective study included 131 eyes of 66 patients with bilateral cataracts that underwent phacoemulsification cataract surgery and Tecnis Symfony IOL implantation. Patients were recruited at the ophthalmology department of Nuestra Señora de Gracia Hospital in Zaragoza, and prospectively evaluated at 1-month, 3-months, and 6-months. Patients were selected according to the guidelines of the general protocol of cataract surgery dictated for our hospital. Inclusion criteria were no alterations or previous ophthalmological surgeries, no dry eye, topographic astigmatisms lower than 1.00 D (total astigmatism, including posterior surface), postoperative corrected visual acuity (CDVA) better than 0.2 logMAR, no intra- or postoperative complications, absence of posterior capsule opacification (PCO) during the study, and a center shift value (distance between corneal apex and center of pupil) lower than 1 mm. The study protocol was approved by the local ethics committee CEICA (Comité de Ética de la Investigación de la Comunidad Autónoma de Aragón) and patients gave written informed consent following the tenets of Declaration of Helsinki.

### Intraocular lens

Tecnis® Symfony® ZXR00 lens is a single-piece, biconvex, hydrophobic acrylic folding lens, with a posterior diffractive surface and an anterior aspherical surface that adds a −0.27 μm spherical aberration to compensate the positive corneal spherical aberration. It also uses a proprietary achromatic diffractive Echelette design that corrects the corneal chromatic aberration for enhanced CS [4]. Its overall diameter is 13.0 mm, and its optical zone diameter is 6.0 mm. The power spectrum available ranges from + 5.0 to + 34.0 D and incorporates an ultraviolet (UV) light-absorbing filter.

### Surgical procedure

Surgery was performed under topical anesthesia by the same experienced surgeon (J.M.L.) and using the same standard phacoemulsification technique. A 2.7 mm clear incision was made at temporal site (180º-0º) using a blade. The capsulotomy size intended by the surgeon was 5.5 mm and the resulting size of capsulotomy next day to the surgery was approximately 5.25 mm. SRK-T, Kane and Barret Universal II formulas were used to calculate the power of the IOL. The target refractive outcome was emmetropia. The selected IOL constant was 119.36. The second eye was intervened 1 month after the first one.

### Study evaluations

All patients underwent a complete preoperative examination that included: exploration of the anterior segment with slit lamp, Goldmann applanation tonometry and posterior pole fundoscopy after pharmacological mydriasis. Optical biometry was performed using IOLMaster 500 (Carl Zeiss Meditec AG, Jena, Germany). AL, anterior chamber depth (ACD), mean keratometry, astigmatism (A) and spherical equivalent (SE) were obtained. Corneal keratometry (K) and aberrations were measured using the Pentacam Scheimpflug camera (OculusWetzlar, Germany). In addition, pupillary size and corneal aberrations were measured using the KR-1 W wavefront analyzer (Topcon Medical Laser Systems, Inc., CA, USA) preoperatively and one month after the implantation of both IOLs.

One-month after the surgery of the second eye, monocular and binocular CDVA and uncorrected distance visual acuities (UDVA) were measured under photopic light conditions (85 cd/m2), using Early Treatment Diabetic Retinopathy Study (ETDRS) charts (ESV-3000 ETDRS System, Vectorvision, Inc.) at 4 m. The procedure was repeated for obtaining VA at intermediate (63 cm) and near (40 cm) distances with best distance correction. Distance VA under environmental mesopic light conditions (6 cd/m2), and using a filter on top of the ETDRS chart, was also measured in this visit.

At 3- months, monocular and binocular distance corrected CS under photopic (85 cd/m2) and mesopic (6 cd/m2) light conditions were measured at 2.5 m with CSV-1000E test using sine wave gratings with different spatial frequencies: 3 cpd (cycles per degree of visual angle), 6 cpd, 12 cpd and 18 cpd. Patients that did not see the first stimuli were assigned a 0 value.

Six months after the surgery, the defocus curve was calculated using powered lenses from −5.00 to + 3.00 D, in intervals of 0.50 D. ETDRS charts were randomly changed 3 times during these procedures to avoid memorization. The first one was used to measure DCVA with 0 defocus. Then, the chart was changed to measure VA using lenses from −5 to −0.50 D, in steps of 0.50D. A third ETDRS chart was used for measuring VA from + 3D to + 0.50D. The range of clear vision (RCV) was obtained monocularly as the magnitude of diopters within the defocus curve in which the best corrected visual acuity (BCVA) was equal or greater than 0.1 logMAR. Patients answered the NEI-RQL-42 questionnaire, which measures vision on daily activities (items 2 to 22), perceived patient’s vision (items 13 to 22), optical corrections (items 23 to 35), and related possible problems (36 to 42). The NEI-RQL-42 score ranges from 0 to 100, 100 representing the best quality of life perceived by the patient. Usually, 13 subitems are calculated based on the following categories: clarity of vision, expectations, near vision, far vision, diurnal fluctuations, activity limitations, glare, symptoms, dependence on correction, worry, suboptimal correction, satisfaction with correction.

### Statistical analysis

Statistical analysis was done in R (version 3.6.1) and RStudio (version 1.2.1335). Data distribution was checked for normality using the Shapiro-Wilks test. Analyses were conducted using generalized estimating equation (GEE) models with an exchangeable working correlation structure to account for correlation between the two eyes from a single participant and using *geepack* package to perform all GEE analyses. *p*-values lower than 0.05 were considered to be statistically significant.

## Results

We included 131 eyes from 66 subjects implanted with Tecnis® Symfony® ZXR00 IOL. One eye was excluded because of epiretinal membrane. Demographic and preoperative biometric measurements are represented in Table 1. Briefly, 31 females and 35 males were included aged between 40 and 76 years (mean age, 64.6 ± 6.7 years old). Mean AL was 23.52 ± 0.85 mm, and mean implanted IOL power was 21.48 ± 2.36 D.

**Table 1:** Demographic and preoperative biometric measurements

	Mean (SD)	Range (min–max)
Demographics		
Age (years old)	64.64 (6.67)	40–76
Gender, n( %female)	31 (47%)	
*Preoperative biometric measurements*		
Refraction		
CDVA (logMAR)	0.28 (0.21)	−0.12–1.00
Spherical equivalent	−0.03 (0.22)	−0.92–0.71
IOLMaster		
Axial length	23.52 (0.85)	21.61–25.50
Anterior chamber depth	3.25 (0.38)	2.40–4.14
Pentacam		
Mean *K*	43.73 (1.30)	40.80–46.50
WFA Z40 (6-mm)	0.39 (0.12)	0.07–0.80
WFA RMS (4-mm)	0.19 (0.08)	0.07–0.47
KR-1 W for 4-mm pupil size		
Total HOA	0.15 (0.06)	0.06–0.53
Spherical	0.05 (0.02)	−0.01–0.11
Astigmatism	−0.68 (0.42)	−2.33–−0.03
Third	0.13 (0.05)	0.02–0.45
Forth	0.08 (0.03)	0.02–0.29
Trefoil	0.09 (0.06)	0.01–0.44
Coma	0.08 (0.04)	0.01–0.19
Tetrafoil	0.03 (0.03)	0.00–0.29
2nd Astigmatism	0.03 (0.02)	0.00–0.08
KR-1 W for 6-mm pupil size		
Total HOA	0.48 (0.27)	0.26–2.31
Spherical	0.27 (0.19)	−0.15–1.73
Astigmatism	−0.53 (0.53)	−4.53–−0.03
Third	0.36 (0.51)	0.07–4.74
Forth	0.36 (0.44)	0.07–4.21
Trefoil	0.24 (0.41)	0.03–3.81
Coma	0.24 (0.31)	0.02–2.83
Tetrafoil	0.13 (0.33)	0.00–3.02
2nd Astigmatism	0.11 (0.26)	0.00–2.37
KR-1 W pupil size		
Scotopic	5.22 (0.94)	2.74–7.41
Photopic	3.44 (0.79)	1.69–5.81

*K* keratometry, *HOA* high order aberrations, *RMS* root mean square, *WFA* wavefront aberration, *Z40* spherical aberration (6-mm zone)

### Postoperative visual acuity and quality of life

**Fig. 1:**
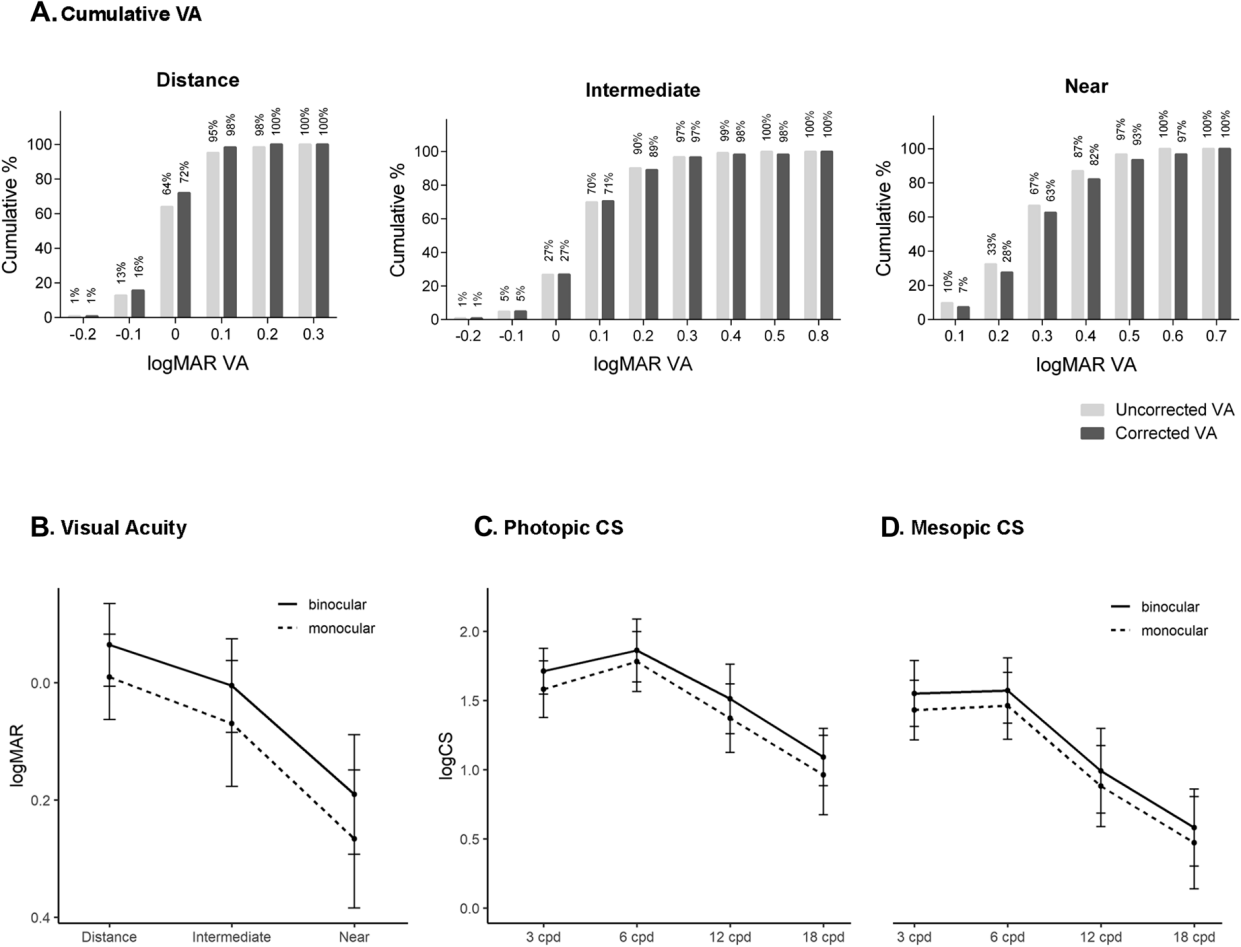
Postoperative visual outcomes after bilateral Tecnis Symfony implantation. **A** Cumulative monocular visual acuities at different distances. Percentage of patients that achieved the distance-corrected logMAR VA at 4 m (distance), 63 cm (intermediate) and 40 cm (near) at 1 month after the implantation of Symfony IOL. **B** Uncorrected mean monocular and binocular visual acuities after 1-month postoperatively. Monocular and binocular corrected contrast sensitivity at 3 months in photopic (**B**) and mesopic (**C**) light conditions. CS, contrast sensitivity; VA, visual acuity

Table 2 and Fig. 1 show the 1-month postoperative measurements. The mean spherical equivalent after surgery was −0.09 ± 0.27 D and UCDVA and CDVA were −0.01 ± 0.07 and −0.02 ± 0.06 logMAR, respectively. The uncorrected visual acuities at intermediate (63 cm) and near (40 cm) distances 1-month after surgery were 0.07 ± 0.11 and 0.27 ± 0.12, respectively, and VAs slightly improved with correction (Fig. 1A). Overall, mesopic distance VAs were lower than photopic distance VAs, but were subject to more improvement after refractive correction (Table 2). The cumulative VAs in Fig. 1A show that, overall, the uncorrected VAs were better at intermediate and near vision but worse at distance, as all patients with postoperative refractive errors—not reaching emmetropia—had myopia or myopic astigmatism (10.6%).

**Table 2:** One-month postoperative refractive and visual outcomes

	Monocular		Binocular	
	Mean (SD)	Range (min–max)	Mean (SD)	Range (min–max)
SE (D)	−0.09 (0.27)	−1.5–0		
Photopic VA (logMAR)				
UDVA	−0.01 (0.07)	−0.2–0.3	−0.06 (0.07)	−0.40–0.10
UIVA	0.07 (0.11)	−0.2–0.44	0.01 (0.08)	−0.16–0.28
UNVA	0.27 (0.12)	0.06–0.58	0.19 (0.10)	−0.06–0.46
CDVA	−0.02 (0.06)	−0.2–0.14	−0.07 (0.07)	−0.40–0.08
DCIVA	0.08 (0.13)	−0.2–0.76	0.00 (0.09)	−0.20–0.24
DCNVA	0.28 (0.12)	0.06–0.58	0.20 (0.10)	−0.06–0.46
Mesopic VA (logMAR)				
UDVA	0.25 (0.10)	0.10–0.64	0.18 (0.08)	0.00–0.36
CDVA	0.24 (0.09)	0.10–0.48	0.17 (0.08)	0.00–0.36
Photopic VA with glare (logMAR)				
UDVA	0.00 (0.08)	−0.18–0.36	−0.06 (0.06)	−0.18–0.10
CDVA	−0.01 (0.07)	−0.18–0.18	−0.07 (0.06)	−0.18–0.08
Mesopic VA with glare (logMAR)				
UDVA	0.25 (0.11)	−0.04–0.52	0.16 (0.10)	−0.06–0.40
CDVA	0.24 (0.11)	−0.04–0.50	0.16 (0.10)	−0.06–0.40

*CDVA* corrected distance visual acuity, *DCIVA* distance corrected intermediate distance visual acuity, *DCNVA* distance corrected near visual acuity, *SE* spherical equivalent, *UDVA* uncorrected distance visual acuity, *UIVA* uncorrected intermediate distance visual acuity, *UNVA* uncorrected near visual acuity, *VA* visual acuity

Table 3 shows CS at different spatial frequencies 3 months after surgery. Altogether, binocular CS was better than monocular CS in all spatial frequencies. Similarly, photopic CS was slightly higher than mesopic CS (Fig. 1B, C). The highest CS was obtained with 6 cpd gratings with a continuous decrease in CS with increasing cycles per degree. All measurements of CS were considered to be within normal ranges.

**Table 3:** Contrast sensitivity results with CSV1000e test at 3 months

	Monocular		Binocular	
	Mean (SD)	Range (min–max)	Mean (SD)	Range (min–max)
Photopic				
3 cpd	1.58 (0.20)	1.17–2.08	1.71 (0.17)	1.34–2.08
6 cpd	1.78 (0.22)	1.38–2.29	1.86 (0.23)	0.70–2.29
12 cpd	1.37 (0.25)	0.61–1.99	1.51 (0.25)	0.40–1.84
18 cpd	0.96 (0.29)	0.13–1.64	1.09 (0.21)	0.64–1.55
Mesopic				
3 cpd	1.43 (0.22)	0.70–2.08	1.55 (0.24)	0.81–2.08
6 cpd	1.46 (0.24)	0.91–1.99	1.57 (0.24)	0.91–2.14
12 cpd	0.88 (0.29)	0.00–1.69	0.99 (0.31)	0.61–1.99
18 cpd	0.47 (0.33)	0.00–1.25	0.58 (0.28)	0.17–1.25

*cpd* cycles per degree

### Impact of refractive error on quality of life

Table 4 shows the subjective quality of vision related to refractive error reported by 53 out of 66 patients 6 months postoperatively. The satisfaction subitem of the questionnaires showed that 96% of patients were very or completely satisfied with the results. At 6 months, 85.5% of patients had little or no difficulties in near vision, and 98% of patients referred optimal vision. Indeed, 90.5% of patients had complete independence of refractive correction, and 34% reported to have no or little difficulty driving. Still, a small percentage of patients reported glare (7.7%) or halos (13.2%) most or all the time.

**Table 4:** NEI-RQL-42 questionnaire results by category

	Mean (SD)	Range (min–max)
Clarity of vision	89.87 (13.78)	39.6–100
Expectation	60.24 (33.20)	0–100
Near vision	84.84 (12.48)	56.25–100
Far vision	80.02 (20.17)	35–100
Diurnal fluctuations	81.78 (16.32)	45.8–100
Activity limitation	95.00 (12.39)	31.3–100
Glare	69.29 (25.24)	0–100
Symptoms	79.79 (16.28)	35.7–100
Dependence on correction	64.05 (29.86)	0–100
Worry	61.43 (28.70)	0–100
Suboptimal correction	97.14 (7.99)	62.5–100
Appearance	92.76 (12.12)	60–100
Satisfaction	91.81 (11.33)	60–100

### Range of clear vision at 6-months

**Fig. 2:**
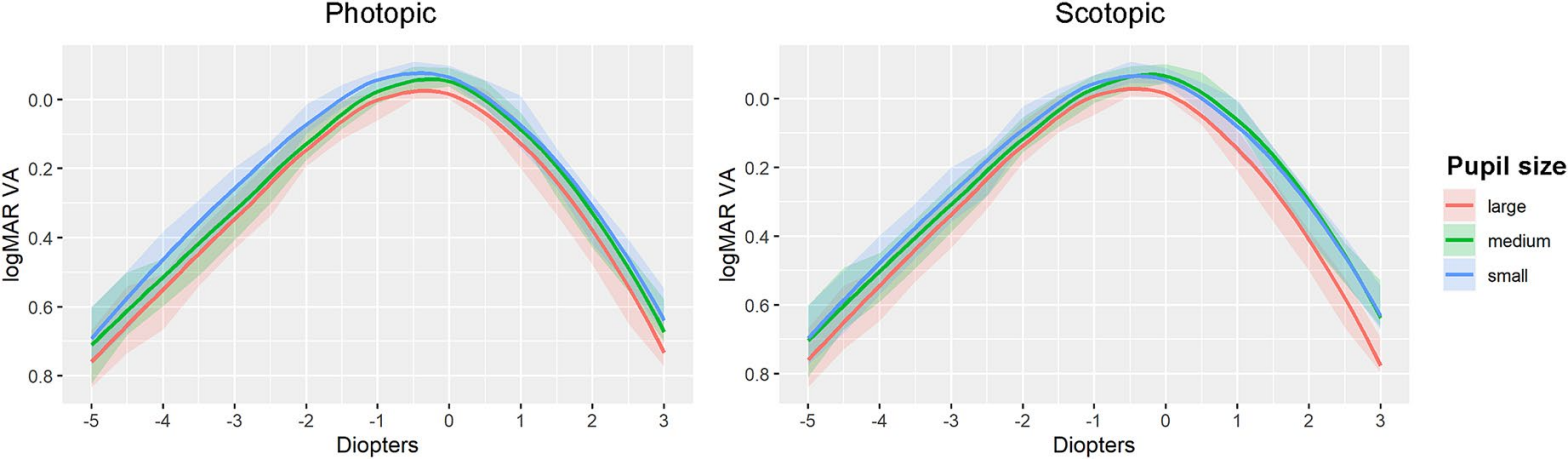
Mean monocular distance-corrected defocus curves at 6 months after bilateral Tecnis Symfony EDoF IOL implantation. Patients were classified into 3 uniform groups according to their pupil size in photopic and scotopic light conditions. Defocus curves are represented for each patient subgroup to show how the VA depends on pupil size. The limits of pupillary diameter for each subset were as follows: in photopic light conditions, small [1.69mm–3.14mm), medium [3.14mm–3.73mm), and large [3.73mm–5.82mm); in scotopic light conditions, small [2.73mm–4.97mm), medium [4.97mm–5.73mm), and large [5.73mm–7.42mm). VA, visual acuity

Six months after the intervention of the second eye, the range of clear vision was calculated. Monocular defocus curve (Fig. 2) showed that CDVA was obtained with −0.18D ± 0.40 defocus lens on average, corresponding to distance vision. The defocus lenses of best VA ranged between + 0.5D and −1.50D. Overall, 90% of the patients obtained an uncorrected binocular logMAR visual acuity better than 0.1 for intermediate vision (-1.5D defocus lens), whereas in near vision, only 17.7% of patients obtained that result. However, 61.2% of patients presented an uncorrected binocular VA of 0.2 logMAR or higher in near vision. The mean range of clear vision was 2.35D ± 0.73D, but it varied considerably among patients, ranging from 0D to 4.5D.

### Preoperative biometric measurements associated with range of clear vision

To identify significant preoperative predictors of the RCV at 6 months, we fitted Generalized Estimating Equations to control from inter-eye intrasubject correlations (Table 5). We found that age was negatively associated with the RCV (*p* = 0.002). The estimated decrease in RCV was −0.05D for every one-unit increase in age. Male sex was significantly associated with broader RCV (*β*: 0.61, *p* = 0.047). Among the ocular biometric parameters, we found that axial length and pupil size were significantly associated with RCV at 6 months, after controlling for the effect of age and sex. The larger the axial length or the pupil size, the narrower the RCV. For axial length, 1-mm increase resulted in 0.35D decrease in RCV (*p* = 0.023). Regarding pupil size, RCV decreased approximately 0.40D per 1 unit increase in pupil diameter (photopic pupil size, *β* = −0.41, *p* = 0.009; scotopic pupil size, *β* = −0.397, *p* = 0.002), after controlling for the effect of age and sex. We failed to find significant associations of preoperative anterior chamber depth, spherical and high-order ocular aberrations for 4-mm pupil size or mean keratometry with postoperative RCV. Nonetheless, we found a significant negative association of spherical aberration, third and fourth order aberrations, and 2nd astigmatism for 6-mm pupil size with RCV in adjusted models.

**Table 5:** General estimating equation models for the association of preoperative variables with postoperative range of clear vision

	GEE			Age and gender adjusted GEE		
	Estimate	SE	*p* value	Estimate	SE	*p* value
Age	−0.053	0.017	**0.002**			
Gender (male)	0.614	0.309	**0.047**			
IOLMaster						
Axial length (mm)	−0.232	0.172	0.177	−0.350	0.154	**0.023**
Anterior Chamber Depth (mm)	0.037	0.397	0.925	−0.429	0.337	0.203
KR-1 W						
Photopic pupil size (mm)	−0.525	0.169	**0.002**	−0.406	0.156	**0.009**
Scotopic pupil size (mm)	−0.428	0.155	**0.006**	−0.397	0.127	**0.002**
*4-mm pupil size*						
Total HOA	0.853	1.741	0.620	0.433	1.220	0.796
Spherical aberration	−5.01	5.78	0.39	−3.562	5.523	0.519
3rd-order	1.874	2.061	0.36	1.074	1.974	0.587
4th-order	−2.671	3.692	0.47	−2.162	3.323	0.513
Trefoil	2.81	2.38	0.24	1.280	2.216	0.564
Coma	−1.863	3.317	0.57	0.142	3.259	0.965
Tetrafoil	−0.865	2.819	0.76	0.068	2.376	0.977
2nd astigmatism	−2.75	6.877	0.69	−4.018	6.481	0.535
*6-mm pupil size*						
Total HOA (6-mm pupil)	−0.269	0.421	0.520	−0.338	0.401	0.399
Spherical aberration (6-mm pupil)	−0.859	0.454	0.059	−0.900	0.421	**0.034**
3rd-order	−0.162	0.088	0.068	−0.183	0.093	**0.049**
4th-order	−0.243	0.118	0.040	−0.285	0.127	**0.025**
Trefoil	−0.174	0.146	0.230	−0.226	0.141	0.107
Coma	−0.188	0.204	0.360	−0.177	0.221	0.425
Tetrafoil	−0.253	0.146	0.083	−0.294	0.151	0.052
2nd astigmatism	−0.416	0.242	0.085	−0.503	0.256	**0.049**
Pentacam						
Mean *K*	0.239	0.131	0.068	0.197	0.120	0.101
WFA Z40	−1.038	1.244	0.400	−0.597	1.252	0.634
WFA RMS	1.272	1.732	0.460	1.694	1.702	0.320

Significant *p*-values are highlighted in bol

*GEE* general estimating equations, *SE* standard error, *HOA* high order aberrations, *K* keratometry, *RMS* root mean square, *WFA* wavefront aberration, *Z40* spherical aberration (6-mm zone)

## Discussion

In this study, we investigated demographic and preoperative biometric measurements associated with 6-months postoperative range of clear vision of eyes implanted with Tecnis® Symfony® ZXR00 IOL after phacoemulsification. Our results indicate that young age, male sex, and smaller axial length and pupil size were associated with wider range of clear vision at 6-months. These results indicate that even in highly selected patients for IOL implantation (less than 1D corneal astigmatism, low ocular aberrations, no ocular pathology), there are demographic and biometric factors that could predict postoperative range of clear vision.

Tecnis® Symfony® ZXR00 is an EDoF IOL that presents a wide range of sharp vision with minimal associated photic phenomena. The results of this study reveal that postoperative UDVA and CDVA were favorable, achieving proper intermediate VAs and enlarged amplitude of pseudo-accommodation. In addition, subjective optimal correction and postoperative patient satisfaction were high. The studies published over the last two years are in line with the current findings, highlighting good visual outcomes after Tecnis Symfony implantation [8–12]. However, most authors agree that near vision might be limited in some patients. Even with distance corrected refraction, it has been observed that some patients achieve good visual acuities (≤ 0.1 LogMAR or 0.8 decimal) as near as 20 cm, whereas others only reach a sharp vision until one meter. Some authors have suggested that targeting a mild myopia in non-dominant eye improves postoperative outcomes [10]. The variability in the range of clear vision with multifocal IOL has been attributed to several factors, but the influence on extended focus IOLs has not been extensively explored. In the current study, we found that younger age was associated with wider range of clear vision, which is in line with studies evaluating apparent accommodation in eyes with a monofocal IOLs [13]. Moreover, we also found that male gender was associated with wider range of clear vision in the defocus curve measured at 6 months. However, it should be noted that the youngest patients were all male, and this fact might have confounded the current results. On the other hand, spherical aberration is known to increase the depth of focus, although it deteriorates CS [14]. Furthermore, other preoperative high order aberrations have also been associated with different postoperative vision measurements, mainly in near vision [15]. However, we only found significant associations between preoperative spherical and high order aberrations and postoperative defocus curve with 6-mm pupil size, and not with 4-mm, which is the effective pupil size in mesopic conditions. This could be because patients were highly selected for the present study and pronounced preoperative aberrations were considered an exclusion criterion, narrowing the variability of aberrations for smaller pupil sizes.

Lastly, preoperative photopic pupil size is critical for multifocal IOL implantation [16], being larger pupil sizes correlated with better distance visual acuity and with worse near visual acuity [17, 18]. Still, the relationship with preoperative pupil size and the postoperative range of clear vision has not been explored in EDoF IOLs. According to our results, preoperative pupil size was negatively associated with the range of clear vision, suggesting that patients with larger pupil sizes presented reduced defocus curves. Lastly, ocular biometric measurements change as a function of age and gender [19–23] and both factors were significantly associated with the outcome of interest. Therefore, all GEE models were adjusted for age and sex. These analyses revealed that axial length was also negatively associated with the range of clear vision. Previous studies have reported that both short axial length and small pupil size predict good near vision after monofocal IOL implantation [23], but their relationship was not explored in EDoF IOLs until now.

This study has several limitations. First, the primary endpoint was the monocular defocus curve measured 6 months after implantation, and no further visual variables were considered, like near and intermediate vision VA or CS or patient satisfaction. However, we believe that the defocus curve is a faithful representation of the dynamic range of clear vision in a single variable. Second, IOL centration was not assessed after IOL implantation, which might have confounded the visual outcomes and the current results. Nevertheless, the EDoF IOLs are more robust against optical quality degradation caused by IOL decentration [24]. Also, the lack of a control group is a major limitation of the current this study, and future works focusing on the performance of EDoF IOLs in comparison with monofocal and multifocal IOLs are needed. Finally, it is important to highlight that we excluded patients with poorer visual performance, like patients with capsular opacification, visual acuity above 0.2 logMAR… so it should be taken into account that our results do not determine the real clinical performance of EDoF lenses. On the other hand, this work has several strengths. The use of GEE overcomes some of the statistical shortcoming of previous studies, in which the intrasubject inter-eye correlation was controlled by including one eye per patients or not controlling at all for this effect. Moreover, as far as we know, this is the first study revealing the association between preoperative demographic and biometric measurements and postoperative range of clear vision after an EDoF IOL implantation and sets the ground for future studies in the field.

In conclusion, the present study demonstrated that age, sex, preoperative pupil size, and preoperative axial length were associated with an enlarged range of clear vision in eyes implanted with Tecnis Symfony ZXR00 IOL. The performance of Tecnis Symfony is expected to be maximized by smaller pupil sizes and axial lengths, and in young patients. Regardless of these variables, Tecnis Symfony provides excellent visual results at distance and at intermediate distances and in different light conditions if patients are carefully selected.
